# Characteristics of Polycystic Ovary Syndrome: A Case Report Investigating the Role of Kapha and Pitta Doshas

**DOI:** 10.7759/cureus.54342

**Published:** 2024-02-17

**Authors:** Ritesh Jadhav, Akash More, Shirish Vaidya, Namrata Choudhary, Shilpa Dutta, Krushnali S Kadu, Gauri Gajabe

**Affiliations:** 1 Anatomy, Datta Meghe Institute of Higher Education & Research, Wardha, IND; 2 Clinical Embryology, Datta Meghe Institute of Higher Education & Research, Wardha, IND; 3 Radiodiagnosis, Datta Meghe Institute of Higher Education & Research, Wardha, IND

**Keywords:** menstrual irregularities, hormonal imbalance, androgen, insulin resistance, ovarian cysts, endocrine disorder

## Abstract

Polycystic ovary syndrome (PCOS) presents complex challenges in diagnosis and treatment due to its multifactorial nature. This case study focuses on a 31-year-old woman exhibiting symptoms of weight gain, irregular menstruation cycles, and hirsutism, leading to a diagnosis of PCOS. Conventional diagnostic criteria and ultrasound confirmation of multiple ovarian cysts supported the diagnosis. By integrating Ayurvedic principles alongside Western medical techniques, this study sought to address imbalances in the Kapha and Pitta doshas, fundamental energies according to Ayurveda, believed to contribute to PCOS symptoms. Clinical findings emphasized the role of Pitta dosha imbalance in inflammation, hormonal irregularities, and excessive body heat, while Kapha dosha imbalance manifested in fluid retention, weight gain, and increased mucus production. A holistic treatment approach was devised, aiming to restore doshic balance while addressing hormonal and metabolic dysregulation. The treatment protocol comprised lifestyle modifications, advocating for a regular exercise regimen focusing on activities enhancing insulin sensitivity and promoting weight loss. Swimming, yoga, and brisk walking were recommended to achieve these goals. Dietary interventions tailored to balance Kapha and Pitta doshas were prescribed, emphasizing nourishing, warming foods low in carbohydrates to prevent weight gain and boost metabolism. Anti-inflammatory foods, such as turmeric and ginger, were incorporated to mitigate inflammation. The integration of Ayurvedic principles alongside Western medicine offered a comprehensive approach to PCOS management, addressing both the root causes and symptoms of the condition. This personalized treatment strategy aimed not only to alleviate immediate symptoms but also to promote long-term health and well-being by restoring doshic equilibrium and optimizing hormonal and metabolic functions. In conclusion, this case study highlights the potential efficacy of combining Ayurvedic and Western medical approaches in the management of PCOS, offering a tailored and holistic treatment paradigm for patients seeking comprehensive care.

## Introduction

Polycystic ovary syndrome (PCOS) is a complex and prevalent endocrine disorder that affects women of reproductive age. Multiple ovarian cysts and hyperandrogenism are among its symptoms, which also include irregular menstrual periods [1]. PCOS is associated with a variety of health issues, such as insulin resistance, obesity, hirsutism (excessive hair growth), and fertility challenges [2]. Other symptoms encompass irregular menstrual cycles and hyperandrogenism (high levels of male hormones), which include many ovarian cysts. In the world of Ayurveda, an ancient medical system originating in the Indian subcontinent, three crucial forces, or "doshas," are said to control health and illness [2]. They are Vita, Pitta, and Kapha doshas. These doshas are supposed to be in balance for overall health, and any imbalance is believed to be a factor in the emergence of several medical disorders [3].

In this case report, we analyze the Pitta and Kapha doshas' potential roles in the origins in detail. To understand how the interaction of these doshas may alter how PCOS symptoms emerge in those who have it, ayurvedic concepts will be applied [4]. This case study aims to further our knowledge of PCOS and examine cutting-edge therapeutic approaches that could complement current treatments or provide specially tailored interventions based on individual dosa imbalances [5]. It achieves this by integrating knowledge from modern medicine with a holistic approach to Ayurveda. It is crucial to note that the scientific and medical communities do not always embrace Ayurvedic theories and methods. Therefore, this report is an exploratory study to encourage further research and dialogue on integrative approaches to managing PCOS and related endocrine disorders [6].

## Case presentation

**Figure 1:**
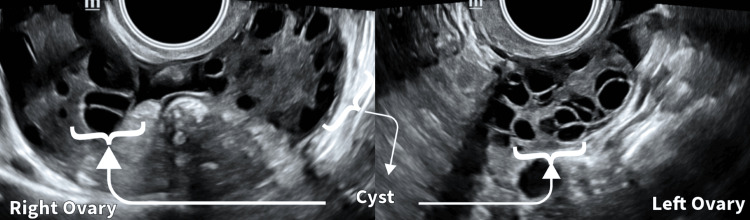
Pre-intervention transvaginal sonography image of the patient's right and left ovaries on day 2 of menstrual cycle. Multiple cysts in the right and left ovaries are depicted.

The patient in this case study is a 31-year-old woman who first complained of weight gain, irregular menstrual cycles, and hirsutism (excessive hair growth). Upon further investigation, the patient was diagnosed with PCOS based on conventional diagnostic criteria and ultrasound confirmation of multiple ovarian cysts. The patient underwent an Ayurvedic evaluation, in addition to traditional diagnostic techniques, to ascertain the possible contribution of the Kapha and Pitta doshas to the appearance of PCOS symptoms. Figure 1 shows the transvaginal sonography image of the patient. According to Ayurveda, doshas are fundamental energies that govern the body and mind. While imbalances can result in various diseases, the balance of these doshas is essential for preserving overall health.

Diagnosis

A 31-year-old woman presented with weight gain, irregular menstruation, and hirsutism. Conventional diagnostic criteria and ultrasound confirmed PCOS. Ayurvedic assessment revealed potential Kapha and Pitta dosha imbalances. The treatment integrates Western and Ayurvedic approaches, targeting hormonal and metabolic issues. Lifestyle adjustments include tailored exercise (swimming, yoga, and brisk walking) to enhance insulin sensitivity and promote weight loss. A personalized diet balances Kapha and Pitta doshas, emphasizing anti-inflammatory foods. Follow-up appointments monitor the progress and adjust treatments for optimal PCOS management.

Clinical findings

While a Pitta dosha imbalance can result in inflammation, hormonal abnormalities, and excessive body heat, a Kapha dosha imbalance may induce fluid retention, weight gain, and excessive mucus production. A comprehensive therapy was created, combining Ayurvedic principles and occidental medical procedures to alleviate the patient's PCOS symptoms effectively. The treatment approach aimed to balance the Kapha and Pitta doshas while treating hormonal and metabolic issues.

Lifestyle

The patient was instructed to begin a regular exercise program emphasizing activities that improve insulin sensitivity and promote weight loss. Swimming, yoga, and brisk walking were suggested as exercises.

Dietary

A personalized diet was prescribed to the patient to balance their Kapha and Pitta doshas. To prevent weight gain and speed up metabolism, the diet comprises nourishing, warming, and low-carb meals. Foods with anti-inflammatory qualities, such as turmeric and ginger, were also promoted.

Follow-up

**Figure 2:**
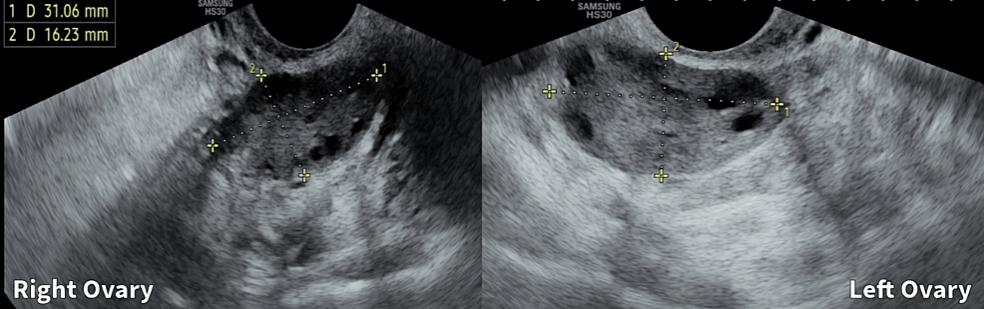
Post-intervention transvaginal sonography image of the patient's right and left ovaries on day 2 of menstrual cycle.

The patient should continue with the prescribed exercise regimen, focusing on swimming, yoga, and brisk walking to improve insulin sensitivity and aid in weight loss. Adherence to the personalized diet, emphasizing nourishing, warming, low-carb meals, and incorporating anti-inflammatory foods like turmeric and ginger, remains crucial. Regular follow-up appointments will monitor progress and adjust the treatment plan as needed for the optimal management of PCOS symptoms. Figure 2 shows the transvaginal sonography image of the patient post-treatment.

## Discussion

A complicated endocrine condition affecting women of reproductive age is PCOS [7]. It is characterized by hormonal imbalances, irregular menstrual cycles, excessive androgen production, and the formation of multiple cysts in the ovaries. While present-day medicine focuses on hormone therapy and lifestyle changes to address PCOS, ancient medical systems, such as Ayurveda, propose a different perspective based on the balance of disease, studying the function of the Kapha and Pitta doshas in the manifestation of PCOS in this case study [4]. Ayurvedic analysis found an excess of Kapha and Pitta doshas, indicating a connection between their imbalance and the development of PCOS. Kapha dosha, weight gain, insulin resistance, and hormonal abnormalities are all common in PCOS patients when they worsen [8]. Pitta dosha, on the other hand, when imbalanced, can cause inflammation and disrupt metabolic processes, further exacerbating the symptoms of PCOS. Based on these findings, a personalized Ayurvedic treatment plan was developed for the patient to restore the balance of Kapha and Pitta doshas [8]. This includes reducing Kapha-aggravating meals and integrating cooling foods for relaxing Pitta. Specific herbal medicines targeting these doshas were also suggested to ease symptoms and address the underlying cause [9].

## Conclusions

This case study underlines the probable value of the Kapha and Pitta doshas in the presentation of PCOS. While further research is needed to establish a conclusive relationship between Ayurvedic principles and PCOS, our findings indicate that adopting Ayurvedic therapies may provide a supplementary strategy to addressing PCOS symptoms. By individualizing treatment plans based on the patient's dosha imbalance, Ayurveda could offer a comprehensive and all-natural complement or alternative to traditional PCOS care. However, it is essential to understand that Ayurveda should not replace evidence-based medical therapies and that a cooperative approach between Ayurvedic practitioners and medical specialists is essential for providing patients with thorough care.
